# Psychometric properties of the Farsi version of the Eating Pathology Symptoms Inventory (F-EPSI) among Iranian University men and women

**DOI:** 10.1186/s40337-022-00587-w

**Published:** 2022-05-09

**Authors:** Reza N. Sahlan, Kerstin K. Blomquist, Lindsay P. Bodell

**Affiliations:** 1 Department of Clinical Psychology, Iran University of Medical Sciences, Tehran, Iran; 2 Department of Psychology, Furman University, Greenville, USA; 3 Department of Psychology, University of Western Ontario, 361 Windermere Road, London, ON N6A 3K7 Canada

**Keywords:** Eating Pathology Symptoms Inventory, F-EPSI, Assessment, Iran

## Abstract

**Background:**

Limited research has validated eating pathology assessments in Iranian men and women. The purpose of the current study was to translate and validate a Farsi version of the Eating Pathology Symptoms Inventory (F-EPSI) in Iranian university students.

**Methods:**

Men (*n* = 279) and women (*n* = 486) completed questionnaires including the F-EPSI.

**Results:**

Confirmatory factor analysis (CFA) indicated that the F-EPSI had an acceptable fit to the data and supported the eight-factor model. The scale was partially invariant across genders. Men reported higher scores on Excessive Exercise and Muscle Building subscales, and women reported higher scores on Body Dissatisfaction and Restricting subscales. The F-EPSI subscales had good 5- to 6-month test–retest reliability. The F-EPSI demonstrated convergent validity with clinical impairment, eating pathology, and body mass index (BMI). Finally, individuals scoring above the Clinical Impairment Assessment (CIA) cutoffs reported higher scores on the F-EPSI subscales, further supporting convergent validity of the scale.

**Conclusion:**

Findings suggest that the F-EPSI will enable researchers to examine eating pathology symptoms among men and women in Iran.

## Plain English summary

The Eating Pathology Symptoms Inventory (EPSI) assesses eight subscales, including muscle building, excessive exercise, body dissatisfaction, binge eating, cognitive restraint, purging, restricting, and negative attitudes toward obesity. This study aimed to assess the psychometric properties of the Farsi version of the EPSI (F-EPSI) among Iranian college men and women. The university students filled out the EPSI and other self-report assessments of eating pathology and related impairment. The results provide support for the F-EPSI’s 8-factor structure among Iranian university students. The F-EPSI was found to be comparable across genders. Convergent validity as well as internal consistency of the F-EPSI were supported. Furthermore, this study supports the reliability of the F-EPSI subscales over time.

## Background

Eating pathology is prevalent among Iranian university students with 21–28% reporting binge eating and 0.9–5% reporting purging behaviors (self-induced vomiting, laxative misuse) [1–3]. Moreover, eating pathology is similar across genders in Iranian samples [1, 2, 4, 5]. Therefore, it is important to have measures that are psychometrically valid and equivalent across genders to improve our understanding of eating pathology in this population.

Existing eating pathology measures that have been translated into Farsi {i.e., Eating Disorder Examination Questionnaire (EDE-Q, [6]), Eating Attitudes Test-26 (EAT-26, [7])} assess symptoms more common among women (e.g., thinness/dietary restraint) than men (e.g., muscularity/exercise; [8]). In Iran, both men and women have similar or higher rates of muscularity/athletic-ideal internalization compared to their counterparts in Western societies [9–12]. Furthermore, eating pathology in Iranian university students was associated with muscularity/athletic-ideal internalization [10] and exercise [13–15], suggesting that muscularity is an important correlate of eating pathology in this population. These findings indicate the need for measures that assess a broader spectrum of eating pathology to be administered in Iranian populations.

The Eating Pathology Symptoms Inventory (EPSI) was validated among men and women in the United States (US) to address the above-mentioned limitations by capturing multiple dimensions of eating pathology cognitions and behaviors, including muscle building, excessive exercise, body dissatisfaction, binge eating, cognitive restraint, purging, restricting, and negative attitudes toward obesity [16]. The 45-item version has demonstrated a robust eight-factor structure in university men and women in the US [17] and China [18] as well as in outpatients with eating disorders in the US [19]. Furthermore, the EPSI was invariant by gender [16] and age [20], suggesting that the EPSI can be compared across genders [21]. Studies from the US and China comparing EPSI scores across genders found that women reported higher scores than men on all subscales except excessive exercise, muscle building, and negative attitudes toward obesity [17, 18, 22]. Such findings are consistent with gender differences in eating pathology typically observed in these populations [1].

With respect to convergent validity, the EPSI subscales have been associated with eating pathology-related clinical impairment, body dissatisfaction, binge eating, purging, and excessive exercise [16–19]. Additionally, the restricting subscale of the EPSI was negatively associated with body mass index (BMI) among general psychiatric outpatients and patients with eating disorders from the US [16]. However, the relationship between BMI and the other EPSI subscales has not been examined [16]. Given that BMI contributes to eating pathology and eating pathology-related clinical impairment in Iranian samples [5, 23], examination of associations between EPSI subscales and BMI is warranted.

Finally, the EPSI subscale scores have demonstrated high internal consistency [16–18] and short-term test–retest reliability (i.e., < 4 weeks; [16, 22]) in US samples. Trolio and colleagues [24] examined test–retest reliability of the EPSI Binge Eating and Restricting subscales up to four months later and reported that the associations were high (*r*s = 0.61–0.74); however, there is no study examining longer-term test–retest reliability of the other EPSI subscales in the literature. Longer-term test–retest reliability is important to support the use of the measure in prospective and treatment outcome research [25].

Given the strong psychometric properties of the EPSI and its potential to capture diverse eating pathology symptoms in both men and women, it would be beneficial to use this assessment to examine eating pathology in Iranian samples. The purpose of the current study was to translate the EPSI into Farsi (F-EPSI) and examine the factor structure, measurement invariance, and other psychometric properties (i.e., convergent validity; internal consistency; test–retest reliability). We postulated the original eight-factor structure of the EPSI would replicate in the Farsi version ([16, 17]; **H1**). Consistent with previous research [16–18, 22], we expected that the F-EPSI factor structure would be invariant across genders **(H2)**, and that women would have higher scores than men on all F-EPSI subscales except for excessive exercise, muscle building, and negative attitudes toward obesity (**H3**). Furthermore, we hypothesized that the EPSI would demonstrate high test–retest reliability (*r* ≥ 0.60) and internal consistency (alpha ≥ 0.70) in both men and women ([17, 18, 22]; **H4**). We also expected that the F-EPSI total score would have a strong correlation (*r* ≥ 0.40) with eating-related clinical impairment, and that the restricting subscale would demonstrate a moderate (*r* ≥  − 0.30) negative correlation with BMI ([16, 19, 26]; **H5**). The associations between the other F-EPSI subscales and BMI were exploratory. We also predicted that individuals scoring above the clinical cut-off for eating related impairment (≥ 16 on the Clinical Impairment Assessment [CIA], [27]) would demonstrate significantly higher scores on the F-EPSI subscales compared to those scoring below the severity cut-off ([23, 27]; **H6**). Finally, we explored associations between the F-EPSI and other measures of eating pathology and impairment across BMI and genders.

## Methods

### Participants

Participants were recruited based on two processes because the COVID-19 pandemic began during data collection. First, university students were recruited from four cities (i.e., Tehran [Capital], Tabriz [Northwest], Mashad [Northeast], Karaj [North]) that are located in diverse geographic regions. Potential participants from a broad range of departments (i.e., Agriculture, Engineering, Psychology, Sociology) were invited to participate in a study that examined psychological issues. Interested students (*n* = 253) completed paper–pencil questionnaires without any remuneration. Due to the pandemic and the lack of students physically on campuses, we were unable to approach students in other departments (i.e., Physics, Chemistry, etc.). Thus, we transitioned to Google drive forms and collected data from 512 more university students. No differences were found between the two methods (paper–pencil method vs. online method) on the F-EPSI subscales mean scores (*p* > 0.05). The final sample (*N* = 765) comprised 279 men and 486 women. Men ranged in age from 18 to 27 years (*M* = 21.2 ± 2.2) and self-reported BMI ranged 15.2–35.0 kg/m^2^ (*M* = 24.3 ± 3.8). Women ranged in age from 18 to 28 years (*M* = 21.10 ± 1.92) and self-reported BMI ranged 15.8–34.2 kg/m^2^ (*M* = 22.9 ± 3.7).

To assess for test–retest reliability, a subset of participants (*n* = 88; 65.9% women) completed the scales 5- to 6-months later. Men ranged in age from 18 to 21 years (*M* = 19.1 ± 1.0) and self-reported BMI ranged 16.2–34.0 kg/m^2^ (*M* = 23.6 ± 4.4). Women ranged in age from 18 to 27 years (*M* = 20.24 ± 1.91) and self-reported BMI ranged 14.7–31.6 kg/m^2^ (*M* = 22.4 ± 3.6). This follow-up sample was younger than the baseline sample (*t* = 6.40, *p* < 0.01). No BMI differences were found. The study was approved by the institutional review board from Iran University of Medical Sciences.

### Measures

#### Demographic information

Participants completed questions regarding age, gender, height, and weight (to calculate BMI kg/m^2^). Although there are limitations to self-reported height/weight, self-reported weight has been correlated highly with objective weight (e.g., *r*s ~ 0.90; [28, 29]).

#### Eating Pathology Symptoms Inventory (EPSI)

The 45-item version of the EPSI is a self-report questionnaire with items rated on a five-point scale ranging from 0 (*Never*) to 4 (*Very often*) [16, 17]. The EPSI assesses eight subscales: Body Dissatisfaction, Binge Eating, Cognitive Restraint, Excessive Exercise, Restricting, Purging, Muscle Building, and Negative Attitudes toward Obesity.

##### Translation procedures

The research team obtained permission from the developer, Dr. Kelsie Forbush, to translate the EPSI into Farsi (F-EPSI) using the parallel back-translation procedure [30–32]. The first author and another independent researcher first translated the EPSI into Farsi. A bilingual colleague familiar with the EPSI compared this version with the original items. A third translator compared these two versions, reconciling linguistic differences. A fourth researcher and clinical psychologist with expertise in eating pathology in Western and non-Western cultures made minor adjustments to ensure conceptual and item equivalence. This final Farsi version was then back-translated by two translators. The original sentences of the scale and back-translated sentences were compared by a bilingual translator in Iran to determine if they held the same meaning, demonstrating semantic equivalence. Finally, the EPSI in its Farsi version was piloted with an independent group of university students (*n* = 39), who were asked to assess the comprehensibility of each item. To do so, this sample was presented with the F-EPSI items in Farsi and asked to circle any word, phrase, or sentence fragment that they found difficult to understand. We found that all the items were understandable within an Iranian context.

#### Farsi-Clinical Impairment Assessment (F-CIA)

The F-CIA [23] assesses functional impairment across personal, cognitive, and social domains due to eating pathology symptoms during the past month. The scale comprises 16 items rated on a 4-point Likert scale ranging from 0 (*Not at all*) to 3 (*A lot*) [23]. Higher scores reflect greater functional impairment, and total scores ≥ 16 suggest clinically meaningful impairment [23]. The F-CIA was invariant across genders and its validity and reliability were supported among Iranian men and women [23]. In this study, Cronbach’s α for the F-CIA total score was 0.95 for both men and women.

#### Farsi-Loss of Control Over Eating Scale (F-LOCES)

The F-LOCES [5] assesses loss of control over eating and consists of three subscales: behavioral, cognitive/dissociative, and positive/euphoric aspects of loss of control over eating. Participants self-reported the frequency of disturbance across the last 28 days on a 5-point Likert scale ranging from 1 (*Never*) to 5 (*Always*), with higher scores reflecting higher pathology [5]. The F-LOCES was invariant across genders and its validity and reliability were supported among Iranian men and women [5]. In this study, Cronbach’s α for the F-LOCES total score was 0.90 for both men and women.

### Analytic plan

There were no missing data on any of the scales administered. We first conducted confirmatory factor analyses (CFA) in MPlus version 8 to test the 8-factor structure of the F-EPSI in the overall sample as well as separately in men and women (**H1**). Consistent with prior research [19, 20], we employed a robust weighted least squares estimator (WLSMV), given the ordinal nature of the data. As suggested in the literature [33, 34], model fit was assessed using the following indices: root mean square error of approximation (RMSEA; < 0.08 indicates a good model fit), the comparative fit index (CFI; > 0.90 indicates an adequate model fit), and the Tucker–Lewis index (TLI; > 0.90 indicates an adequate model fit).

Multi-group CFA (MGCFA) was used to examine measurement invariance across genders (**H2**; [35]). Configural invariance evaluated the extent to which the factor structure of the F-EPSI was similar across genders. Metric invariance evaluated the extent to which factor loadings were equivalent across genders, and scalar invariance evaluated the extent to which the F-EPSI items had similar intercepts in men and women. We used change in model fit indices between the configural and metric models and between the metric and scalar models as evidence of measurement invariance, based on the following criteria: ΔRMSEA < 0.015, ΔCFI < 0.002, and ΔTLI < 0.01 [36–38]. Although we also examined the chi-square difference test in Mplus (i.e., DIFF test), these indices are sensitive to complex models [39, 40]; thus, interpretation of measurement invariance was based on the other metrics noted above. Upon establishing measurement invariance across genders using MGCFA, independent sample *t*-tests examined mean differences of the F-EPSI subscales scores across genders (**H3**).

Cronbach’s alpha and McDonald’s ω assessed internal consistencies (**H4**; [41]). The following cut-offs were used to indicate good internal consistency: Cronbach’s alphas of the F-EPSI subscales scores were determined to be good at α ≥ 0.70 [42]; McDonald’s ω [41] at ≥ 0.70 was determined to be acceptable [43]. Interclass correlations (ICCs) examined test–retest reliability of the EPSI subscales scores over a 5- to 6-month period (**H4**; [44]). ICCs were calculated using a two-way random with absolute agreement for single measures [22]. Based on guidelines developed by Cicchetti [45], ICC values < 0.40 are considered “poor,” 0.40 to 0.59 “fair,” 0.60 to 0.74 “good,” and ≥ 0.75 are “excellent”.

To establish convergent validity of the F-EPSI (**H5**), Pearson’s correlations examined associations between F-EPSI subscales and the F-CIA total score as well as between the F-EPSI Restricting subscale and BMI. In addition, Pearson’s correlations were used to explore the associations between the other F-EPSI subscales and BMI as well as between the F-EPSI subscales and the F-LOCES scores. According to Cohen [26], correlation coefficients of 0.10, 0.30, and 0.50 are considered to be small, medium, and large correlations, respectively. To further assess convergent validity, independent sample *t*-tests compared university samples scoring above (≥ 16) and below (< 16) the proposed CIA severity cutoff [23, 27] on the F-EPSI subscales (**H6**). Lastly, Fisher’s *z* test compared the associations between F-EPSI subscales with F-CIA, F-LOCES, and BMI across genders. We used SPSS 25.0 for these analyses.

## Results

### Confirmatory factor analysis and tests of invariance

In support of **H1**, the original 8-factor structure of the EPSI demonstrated adequate fit to the data in the overall sample with RMSEA = 0.056 (95% CI 0.054–0.059), CFI = 0.914, and TLI = 0.907. Findings were similar in men and women (see Table 1). No men endorsed response option 4 (always) for item 28 “I did not notice how much I ate until after I had finished eating;” thus, response options “3” and “4” for this item were collapsed for women when conducting tests of measurement invariance across groups. Model fit statistics and change in fit values from the multi-group CFAs are included in Table 1. The F-EPSI factor structure demonstrated good fit in both men and women, providing evidence for configural invariance. Change in fit indices between the configural and metric models provided support for metric invariance, suggesting that factor loadings were equivalent across genders, which supported **H2**. Although there was evidence of scalar invariance across genders based on change in RMSEA and TLI, scalar invariance was not indicated based on change in CFI. These findings are consistent with recent work examining measurement invariance across adolescents and adults [20] and provide support for similar factor structure and factor loadings across genders; however, there may be variance at the level of the intercepts (see Table 1).

**Table 1:** Model fit statistics and measurement invariance across genders

	Chi-square	df	DIFF test (df)	RMSEA (ΔRMESA)	CFI (ΔCFI)	TLI (ΔTLI)
Men	1666.60	917	–	0.054	0.935	0.929
Women	2180.15	917	–	0.053	0.922	0.916
Configural invariance	3829.94	1834	–	0.053	0.926	0.920
Metric invariance	3883.30	1871	80.54 (37)**	0.053 (0)	0.925 (.001)	0.921 (.001)
Scalar invariance	3908.12	1997	162.68 (126)*	0.050 (.003)	0.929 (.004)	0.930 (.009)

*p < .05; **p < .001

### Gender differences

In support of **H3**, women endorsed significantly higher scores than men on the Body Dissatisfaction and Restricting subscales (see Table 2). Furthermore, men had significantly higher scores than women on the Excessive Exercise and Muscle Building subscales. However, contrary to our hypothesis, there was no mean difference on Negative Attitudes toward Obesity across genders. Lastly, no gender differences emerged for the remaining F-EPSI subscales.

**Table 2:** Means (standard deviations) and t test by gender and by total CIA scores for the F-EPSI subscales

Subscales	Men (*n* = 279)	Women (*n* = 486)	*t*	*p*	Cohen’s *d*^a^
	*M* (*SD*)	*M* (*SD*)			
Body dissatisfaction	7.17 (5.56)	8.72 (6.43)	3.36	.001	.26
Binge eating	6.38 (5.11)	5.92 (5.08)	1.18	.24	.09
Cognitive restraint	3.96 (2.36)	4.15 (2.64)	.97	.33	.08
Purging	1.90 (3.61)	1.92 (3.39)	.12	.90	.00
Restricting	7.14 (4.20)	8.26 (4.79)	3.24	.001	.25
Excessive exercise	6.93 (4.54)	5.52 (3.90)	4.51	.001	.33
Negative attitudes toward obesity	6.64 (4.67)	7.0 (4.85)	1.03	.31	.08
Muscle building	3.17 (3.93)	2.24 (3.22)	3.55	.001	.26

	CIA < 16 (*n* = 586)	CIA ≥ 16 (*n* = 179)			
Body dissatisfaction	6.84 (5.45)	12.46 (6.45)	11.56	.001	.94
Binge eating	4.79 (4.20)	10.34 (5.45)	14.37	.001	1.14
Cognitive restraint	3.90 (2.56)	4.66 (2.41)	3.65	.001	.31
Purging	1.20 (2.56)	4.25 (4.80)	11.03	.001	.79
Restricting	7.43 (4.70)	9.23 (4.05)	4.63	.001	.41
Excessive exercise	5.47 (4.07)	7.88 (4.09)	6.91	.001	.59
Negative attitudes toward obesity	6.15 (4.61)	9.25 (4.58)	7.92	.001	.67
Muscle building	2.0 (2.97)	4.45 (4.42)	8.52	.001	.65

^a^Effect size (Cohen’s *d*)

### Internal consistency and intercorrelations among subscales

In support of **H4**, the F-EPSI subscales demonstrated good internal consistency based on both Cronbach’s alphas and McDonald’s ω. All of the F-EPSI subscales were significantly, positively intercorrelated for men and women, with small-to-large correlations (see Table 3).

**Table 3:** Internal consistency and intercorrelations among F-EPSI subscales in university men (n = 279) and women (n = 486)

F-EPSI subscales	Cronbach’s Alphas	McDonald’s ω	1	2	3	4	5	6	7	8
1. Body dissatisfaction	.86/.87	.86/.87	–	.59***	.29***	.43***	.45***	.39***	.47***	.38***
2. Binge eating	.86/.87	.86/.87	.51***	–	.30***	.59***	.52***	.44***	.55***	.53***
3. Cognitive restraint	.71/.72	.72/.74	.28***	.19***	–	.25***	.23***	.50***	.33***	.24***
4. Purging	.90/.83	.90/.83	.37***	.51***	.29***	–	.36***	.25***	.33***	.60***
5. Restricting	.73/.77	.74/.77	.26***	.22***	.25***	.18***	–	.32***	.47***	.39***
6. Excessive exercise	.83/.79	.83/.79	.35***	.33***	.49***	.39***	.30***	–	.41***	.47***
7. Negative attitudes toward obesity	.82/.83	.82/.83	.48***	.43***	.27***	.28***	.27***	.31***	–	.37***
8. Muscle building	.88/.84	.88/.85	.26***	.41***	.12**	.53***	.32***	.38***	.20***	–

The values of Cronbach’s Alphas and McDonald’s ω before the slash are for men and the values after the slash are for women. Intercorrelations above the diagonal are for men and below the diagonal are for women

*F-EPSI* Farsi version of the Eating Pathology Symptoms Inventory

**p < .01; ***p < .001

### Test–retest reliability

In support of **H4**, the F-EPSI subscales demonstrated good-to-excellent test–retest reliability in men (all ICCs ≥ 0.60) and women (all ICCs ≥ 0.76) (see Table 4).

**Table 4:** Test–retest reliability in men (n = 30) versus women (n = 58) for F-EPSI subscales

F-EPSI subscales	Men		Women	
	ICC	ICC CI	ICC	ICC CI
Body dissatisfaction	.89	[.78, .95]	.78	[.65, .86]
Binge eating	.74	[.52, .87]	.81	[70, .88]
Cognitive restraint	.60	[.31, .78]	.76	[.63, .85]
Purging	.71	[.48, .85]	.92	[.85, .95]
Restricting	.76	[.56, .88]	.77	[.64, .85]
Excessive exercise	.82	[.66, .91]	.81	[.71, .88]
Negative attitudes toward obesity	.63	[.36, .80]	.76	[.63, .85]
Muscle building	.66	[.40, .82]	.84	[.74, .90]

*ICC* Intraclass correlations, *CI* Confidence interval

### Convergent validity

In support of **H5**, all the F-EPSI subscales demonstrated significant, positive associations with the F-CIA scores. The Restricting subscale had a negative association with BMI in women, but contrary to hypotheses, this subscale was not associated with BMI in men.

Our exploratory analyses indicated that the Muscle Building subscale negatively correlated with BMI in women, but it was not associated with BMI in men. The remaining F-EPSI subscales were positively associated with BMI in men and women. Furthermore, the EPSI subscales were significantly correlated with the F-LOCES scores (see Table 5). Approximately 23% (*n* = 179) of participants reported F-CIA total scores above the F-CIA cutoff point (≥ 16; 27). In support of **H6**, individuals with high clinical impairment (F-CIA ≥ 16) reported significantly higher scores on all the F-EPSI subscales (*p* < 0.001; Table 2).

**Table 5:** Pearson correlations between F-EPSI subscales and eating pathology-related measures in men (n = 279) and women (n = 486)

	Body dissatisfaction	Binge eating	Cognitive restraint	Purging	Restricting	Excessive exercise	Negative attitudes toward obesity	Muscle building
CIA-T	.46***/.40***	.55***/.58***	.16**/.13**	.47***/.40***	.38***/.18***	.26***/.34***	.36***/.37***	.40***/.33***
BMI	.21***/.40***	.16**/.27***	.28***/.27***	.22***/.22***	.03/ − .13**	.18**/.21***	.18**/.20***	− .06/ − .11*
LOCES-T	.46***/.50***	.73***/.77***	.32***/.24***	.43***/.35***	.37***/.15***	.32***/.32***	.41***/.41***	.30***/.24***

The values of Pearson correlations before the slash are for men and the values after the slash are for women

*CIA-T* Clinical Impairment Assessment-Total score, *BMI* body mass index, *LOCES-T* Loss of Control Over Eating Scale-Total score

*p < .05; **p < .01; ***p < .001

### Comparison of correlations across genders

A series of Fisher’s *z* tests revealed that the correlations between the Restricting subscale with F-LOCES (*z* = 3.15, *p* < 0.001) and F-CIA (*z* = 2.89, *p* < 0.01) were stronger for men than women; the correlation between Body Dissatisfaction subscale with BMI (*z* =  − 2.79, *p* < 0.01) was stronger in women than men. The strengths of the correlations among other variables did not differ significantly by gender.

## Discussion

This study translated the EPSI into Farsi and demonstrated the scale’s validity and reliability among Iranian university students. Consistent with previous studies in the US [17, 19] and China [18], results support the eight-factor structure of the F-EPSI with high internal consistencies across subscales. The current study adds to the literature by demonstrating that the F-EPSI subscale scores were stable over a 5- to 6-month period [44] and indicates the suitability of the scale for use in longitudinal studies in Iran.

Consistent with our hypotheses and with previous research in the US [16], the F-EPSI factor structure was largely equivalent across genders, demonstrating evidence for configural and metric invariance, as well as some evidence for scalar invariance. Scalar variance (i.e., variance in item intercepts) between men and women implies that one group may find the items “easier” to endorse; thus, differences in scores may not reflect true differences in the level of the latent construct. Collectively, our findings suggest that F-EPSI is appropriate to use for exploring gender differences. Consistent with previous research [17], women endorsed significantly higher scores than men on the Body Dissatisfaction and Restricting subscales, and men reported higher Excessive Exercise and Muscle Building than women. Importantly, no gender differences emerged for Binge Eating, Cognitive Restraint, and Purging subscales, suggesting that many forms of eating pathology (i.e., restraint, purging, binge eating) are similar across genders. Contrary to previous studies [17, 18], Negative Attitudes toward Obesity subscale was not different across genders. One explanation is that weight-related stigma is not limited to men or women in Iran, but rather both report similar levels of weight-related stigma.

The F-EPSI subscales were associated with impairment (i.e., F-CIA scores) and loss of control over eating (i.e., F-LOCES). Consistent with US samples [16], the Restricting subscale was *negatively* associated with women’s BMI, suggesting that the Restricting subscale may capture actual caloric restriction in women. However, the Restricting subscale was not associated with BMI in men, indicating that shape/weight preoccupation irrespective of body weight might contribute to eating-related restriction in Iranian men [4]. Additionally, the Muscle Building subscale was not associated with men’s BMI, which aligns with a previous study in Iran [10]. This finding may suggest that men, irrespective of BMI, tend to use supplements to build muscularity. In contrast, the Muscle Building subscale was negatively associated with women’s BMI, such that lower BMI was related to more muscle building behaviors, which is consistent with the thin ideal for women including aspects of muscularity. It could also suggest that women with higher BMI may be motivated to lose weight via more socially acceptable forms of weight loss (e.g., shape/weight-related exercise). Interestingly, associations with other F-EPSI subscales and BMI were significant in men and women, suggesting that BMI may be a correlate of eating pathology in Iranian university students. Collectively, these findings support the convergent validity of the F-EPSI.

Inconsistent with a previous study in the US [17], we found a stronger association between the Restricting subscale and both the F-CIA and F-LOCES in men compared to women. One possible explanation is that dietary restriction may represent a more impairing form of eating pathology in men compared to women. For example, restrictive eating may be seen as more normative in women and thus less strongly associated with clinical impairment. On the other hand, the correlation between Body Dissatisfaction and BMI was stronger in women than men, which aligns with the literature [46], and may reflect gender-related body ideals. For example, women of higher body weights may be more dissatisfied with their body than men of higher body weights if cultural values related to thinness are stronger for women than men. The correlations between the other F-EPSI subscales and F-CIA, F-LOCES, and BMI were not significantly different between men and women, indicating that clinical impairment, loss of control over eating, and BMI are implicated similarly in men and women’s scores on the F-EPSI.

In further support of the F-EPSI’s convergent validity, individuals scoring above the CIA clinical cutoff reported higher eating pathology, particularly purging. Indeed, 23.4% of students in the current study reported eating-related clinical impairment, which is higher than previous studies in Iranian adolescents (i.e., 17.4%; [23]) and Western university samples (i.e., 11%; [47]). Findings highlight the impact of eating pathology on young adults in Iran, and the importance of its prevention and treatment in this understudied population.

### Strengths and limitations

The strengths include the relatively large sample and the diverse range of eating pathology symptoms assessed by the EPSI. However, the results may not generalize to other samples, such as patients with eating disorders and adolescents. Thus, examining the F-EPSI among those groups would be an important next step. Finally, measures were not available for evaluating the discriminant validity of the F-EPSI in the current sample. Therefore, future studies should examine discriminant validity of the scale in other samples.

## Conclusion

The F-EPSI is a psychometrically valid and reliable scale that can be used in both research and therapeutic settings. It assesses eating pathology in both women (e.g., thinness/dietary restraint) and men (e.g., muscularity/exercise), is invariant across genders, and can be used to reliably measure change in eating pathology over time in longitudinal studies.

## Data Availability

The data that support the findings of this study are available on request to the first author.

## Abbreviations

US: United States
EPSI: Eating Pathology Symptoms Inventory
CIA: Clinical Impairment Assessment
LOCES: Loss of Control Over Eating Scale
BMI: Body mass index
EDE-Q: Eating Disorder Examination Questionnaire
EAT-26: Eating Attitudes Test-26
CFA: Confirmatory factor analysis
MGCFA: Multi-group CFA
WLSMV: Weighted least squares estimator
RMSEA: Root mean square error of approximation
CFI: Comparative fit index
TLI: Tucker–Lewis index
ICCs: Interclass correlations
